# Clinical and functional outcomes of isolated posterior cruciate ligament reconstruction in patients over the age of 40 years

**DOI:** 10.1186/s12891-022-05151-w

**Published:** 2022-03-05

**Authors:** Chia-Hung Liu, Chih-Hao Chiu, Shih-Sheng Chang, Wen-Ling Yeh, Alvin Chao-Yu Chen, Kuo-Yao Hsu, Chun-Jui Weng, Yi-Sheng Chan

**Affiliations:** 1 Department of Orthopedic Surgery, Chang Gung Memorial Hospital, Linkou, Taiwan; 2 Comprehensive Sports Medicine Center, Chang Gung Memorial Hospital, Linkou, Taiwan

**Keywords:** Posterior cruciate ligament, Reconstruction, Older patients, Clinical outcomes

## Abstract

**Background:**

To assess clinical and functional outcomes of patients aged 40 years or older receiving PCL reconstruction surgery.

**Methods:**

All patients older than 40 years with isolated PCL rupture who underwent PCL reconstruction surgery were enrolled into the retrospective study. Associated meniscal injuries, osteochondral lesions, postoperative complications, and the rate of return to the preinjury level of activity were extracted. Outcomes included International Knee Documentation Committee (IKDC) subjective score, Lysholm score, and Tegner activity score. The minimal clinically important difference (MCID) and patient acceptable symptom state (PASS) were used to evaluate the clinically relevant value of PCL reconstruction in this population.

**Results:**

In total, 41 patients with a mean age of 51.7 years were included. The mean follow-up time was 32.8 months. Associated lesions included meniscal injuries (48.8%) and osteochondral lesions (97.6%). Improvement in the IKDC score (from 46.5 preoperatively to 79.0 postoperatively, *p* < 0.0001), Lysholm score (from 65.5 to 88.3, *p* < 0.0001), and Tegner activity score (from 2.3 to 4.0, *p* < 0.0001) was recorded. The clinically relevant value based on the MCID showed that 34 of 41 patients (82.9%) had a ΔIKDC score exceeding 16.8; all patients (100%) showed a ΔLysholm score exceeding 8.9; and 35 of 41 patients (85.4%) showed a ΔTegner activity score exceeding 0.5. Regarding the PASS, none of the patients had an IKDC score exceeding 75.9 preoperatively, whereas 27 of 41 patients (65.9%) had a score of more than 75.9 postoperatively. All patient had ≥ grade II knee instability preoperatively. Postoperatively, 36 patients (87.8%) had no significant joint translation, and 5 patients (12.2%) had grade I instability. Twenty-one patients (51.2%) returned to their preinjury level of activity. Five patients (12.2%) developed Ahlbäck grade I radiographic osteoarthritis. No rerupture or other major perioperative complications were reported.

**Conclusions:**

PCL reconstruction is a reliable surgery for middle-aged patients suffering from persistent instability even after failed conservative treatment, with significant improvement in patient-reported outcomes that exceeded MCID in the majority of patients, restoration of subjective instability, and approximately half of the patients returned to preinjury activity levels.

**Level of evidence:**

Level IV, therapeutic case series.

## Background

Posterior cruciate ligament (PCL) injury is one of the most common soft-tissue injuries of the knee. Previous studies have revealed that patients who suffer from isolated PCL injury have satisfactory subject outcomes and acceptable functional scores after receiving prolonged conservative care [1–3]. Therefore, most experts would agree treatment of asymptomatic PCL injury is nonoperative management.

Despite this, recent studies have shown that conservative treatment can increase the risk of developing osteoarthritis, which may result from high-grade PCL laxity or progression of meniscus tears [1, 4, 5]. Shelbourne et al. [1] followed 68 isolated PCL-injured patients treated nonoperatively for a mean of 17 follow-up years and found that the patients remained active and noted good subjective scores; however, this study reported that 23% and 41% of patients developed osteoarthritis after 7 and 14 years, respectively, and 11% of the patients experienced moderate to severe osteoarthritis. These results have raised concerns among surgeons regarding early surgical intervention to restore stability and to prevent further OA changes, especially in high grade symptomatic tears [6].

Surgical treatment with PCL reconstruction is an effective method for patients with significant PCL laxity and painful symptoms [7–13]. Many studies have reviewed clinical and functional outcomes of PCL reconstruction focusing on comparison different surgical techniques (single- versus double-bundle or tibial inlay versus transtibial, with and without remnant preservation), graft sources (autograft versus allograft), or isolated and multiligament injuries. For anterior cruciate ligament (ACL) reconstruction, studies have investigated surgical outcomes in older age groups, and most results concluded that patients older than 40 years [14, 15] and even over 50 years [16, 17] can achieve satisfactory outcomes, with good symptomatic relief, restoration of function and return to sport rate after reconstruction.

Considering the increasing demand for higher quality of life and the more active lifestyle of today’s elderly population with a longer life expectancy, the appropriate treatment for PCL injury is of increasing importance. We wondered if older patients could benefit from PCL reconstruction surgery. However, there is a paucity of reports on surgical outcomes of isolated posterior cruciate ligament reconstruction in patients older than 40 years.

The purpose of this study was to assess the clinical and functional outcomes, including the return to preinjury activity level and the progression of arthritis, in patients aged 40 years or older after PCL reconstruction. We hypothesized that patients aged 40 years or older would show good to excellent clinical and functional outcomes after PCL reconstruction.

## Methods

**Fig. 1:**
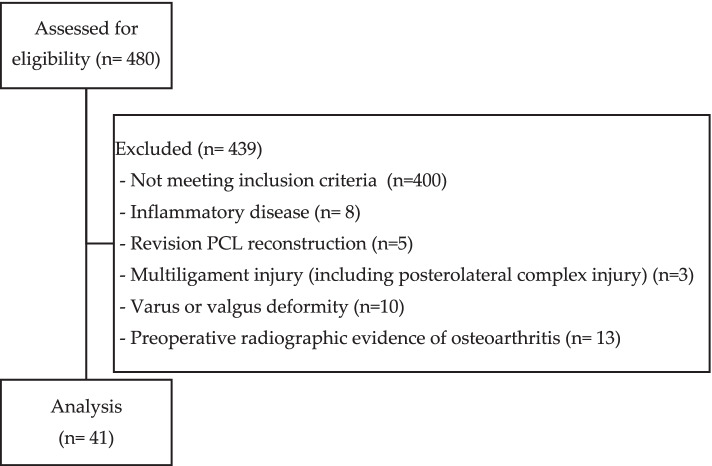
Flow chart of criteria regarding enrollment and exclusion

This retrospective study was conducted at a medical center and was approved by the ethical committee at our hospital. Deidentification of patient data was required prior to conducting the study. Two hundred and eighty (*n* = 480) patents were accessed for eligibility. All patients older than 40 years with a clinical and imaging diagnosis of isolated PCL rupture who underwent PCL reconstruction surgery in our hospital between 2012 to 2017 were recruited into the study. The inclusion criteria were as follows: (1) aged 40 years or older, (2) follow-up period longer than two years, and (3) an uninjured contralateral leg. Eighty patients (*n* = 80) met all the above criteria. Patients with inflammatory disease (*n* = 8), revision PCL reconstruction (*n* = 5), multiligament injury (including posterolateral complex injury) (*n* = 3), varus or valgus deformity (*n* = 10), or preoperative radiographic evidence of osteoarthritis (Ahlbäck grade ≥ 2) (*n* = 13) were excluded from the study. Forty-one patients (*n* = 41) were analyzed. Criteria regarding enrollment and exclusion showed in Fig. 1. Diagnosis was made through clinical symptoms, physical examination and magnetic resonance imaging (MRI) study according to our standard knee MRI protocol (sagittal, axial, and coronal fat-suppressed fast spin-echo T2-weighted images and sagittal fat-suppressed spin-echo proton-density images).

All patients were initially treated nonsurgically. Protected weight-bearing for 2–4 weeks and quadriceps strengthening were indicated for patients with partial tear. And patients with full tear received brace protection in full extension for 2–4 weeks with ROM exercises at least 2 times a day. Surgical treatment was indicated when patients with functional demands presented with instability symptoms, such as giving out sensation, popping or snapping sound, and feeling of looseness in daily or sports activities after failure of nonsurgical treatment.

All 41 patients were treated in our hospital by 3 experienced orthopedic doctors using the transtibial technique. Single-bundle PCL reconstruction surgery was performed on 33 patients, and double-bundle PCL reconstruction surgery was performed on the other 8 patients. We used autologous hamstring tendon grafts for reconstruction in all patients. Twenty-four patients received graft fixation with an interference screw (HA Interference Screw; Smith & Nephew) on both the femoral and tibial sides, whereas seventeen patients received graft fixation with an interference screw on the tibial side and suspensory button fixation (EndoButton; Smith & Nephew) on the femoral side.

All patients followed the same postoperative rehabilitation protocol. Brace protection in full extension was used immediately postoperatively and unlocked for gradual passive range of motion at 2 weeks postoperatively. Partial weight-bearing ambulation with crutches assistance was suggested for 4 to 6 weeks, followed by full weight bearing. Isometric quadriceps exercises were executed immediately after the operation, and a therapeutic exercise program was set up and conducted by a physiatrist. Patients were allowed to participate in low-levels sports activities, such as jogging and swimming, 6 months after surgery and in high-level sports activities, such as contact sports or those involving pivoting movements, 9 months after surgery. For safe return to preinjury activity levels, clinical examinations with knee and ankle joint range of motion, muscle girth and muscle power of quadriceps compared with healthy side, posterior drawer test, and hop test were checked prior to moving forward to higher activity levels.

The preoperative clinical evaluation and the last follow-up were performed by the surgeon. Objective clinical examination consisted of the posterior drawer test, posterior sag sign assessment, McMurray test, and range of motion. The grade of instability was assessed by Petrie and Harner classification [18]. Preoperative MRI was reviewed to confirm that no other associated ligamentous injuries were present. Radiographs were taken preoperatively and at the last OPD follow-up for determination of the stage of osteoarthritis according to Ahlbäck classification by the attending radiologist. Functional outcomes, including the International Knee Documentation Committee (IKDC) subjective score, Lysholm score, and Tegner activity level, were measured preoperatively at the time of admission to the hospital and at the final follow-up through interviews of the patients by an independent orthopedic doctor. Minimal clinically important difference (MCID) and patient acceptable symptom state (PASS) for the IKDC score, Lysholm score, and Tegner activity score were used to confirm whether the outcomes were clinically relevant and significant [19]. MCID was determined by Distribution-based methods, and PASS was defined by previously published threshold [20] Table 1 lists the defined values.

**Table 1:** Defined Values of MCID and PASS

	**MCID**	**PASS**
IKDC score	16.8	75.9
Lysholm score	8.9	NA
Tegner score	0.5	NA

*IKDC* International Knee Documentation Committee, *MCID* minimal clinically important difference, *PASS* patient acceptable symptom state, *NA* not available

For statistical analysis, the paired- samples t test was used to compare changes in the functional outcome. The analysis was carried out using SPSS software (version 22.0; SPSS, Armonk, NY) by an independent statistician. The level of statistical significance was set at *P* < 0.05.

## Results

Of the 41 patients in this study, the mean age at the time of the survey was 51.7 years (range, 40–68 years). There were 23 males and 18 females. The mean body mass index (BMI) was 24.4 kg/m^2^ (range, 16.3–30.2 kg/m^2^). The mean follow-up period was 32.9 months (range, 24–71 months). Patient demographics are listed in Table 2.

**Table 2:** Demographic Characteristics of Patients

	**Data**
No. Of patients	41
Sex: male/female, n	23/18
Side of surgery: left/right, n	18/23
Mean age (range), yr	51.7 (40–68)
Mean BMI (kg/m^2^)	24.4 (16.3–30.2)
Mean follow-up time (range), mo	32.9 (24–71)
Mean time from injury to index surgery (range), mo	4.8 (1–12)
Positive posterior drawer test, n (%)	41 (100)
Positive posterior sag sign, n (%)	30 (73)
Positive McMurray test, n (%)	16 (39)
Preoperative Ahlbäck classification, n (%)	
Grade 0	23 (56.1)
Grade 1	18 (43.9)
Grade 2	0
Grade 3	0
Grade 4	0
Preoperative instability as Petrie and Harner classification, n (%)	
Normal	0
Grade I	0
Grade II	10 (24.4)
Grade III	31 (75.6)

The most common mechanism of injury was traffic accident (24 patients, 58.5%), followed by sprain/falling injury during daily activity (9 patients, 22.0%), sport injury (5 patients, 12.2%), and work-related activities (3 patients, 7.3%).

The mean time from injury to surgery was 4.8 months (range, 1–12 months). At the time of preoperative radiographic survey, 23 patients (56.1%) had no finding of radiographic osteoarthritis, and 18 patients (43.9%) had grade 1 osteoarthritis according to the Ahlbäck classification. At the time of preoperative physical examination, 10 patients (24.4%) had grade II, and 31 patients (75.6%) had grade III joint translation according to the Petrie and Harner classification. Meniscal tears were found in 20 patients (48.8%) at arthroscopy. Of the 20 patients, 9 patients (22%) had horizontal tear patterns which are characteristic of degenerative meniscal tear, whereas the others 11 patients (26.8%) had traumatic meniscal tear. The tear was located at the medial meniscus in 10 patients, lateral meniscus in 7 patients, and both the medial and lateral meniscus in 3 patients. Five patients were treated with meniscal repair, and 15 patients were treated with partial meniscectomy. Cartilage lesions were defined as Outerbridge grade ≥ 1 and were found in 40 patients (97.6%). Five patients were treated with microfracture. Detailed intraoperative findings of associated injuries are listed in Table 3.

**Table 3:** Intraoperative Findings of Associated Injuries

Lesion	n (%)
Cartilage lesion	40 (97.6)
Grade 1	21 (51.2)
Grade 2	14 (34.1)
Grade 3	4 (9.8)
Grade 4	1 (2.4)
Meniscal lesion	20 (48.8)
Medial	10 (24.4)
Lateral	7 (17.1)
Both	3 (7.3)

**Fig. 2:**
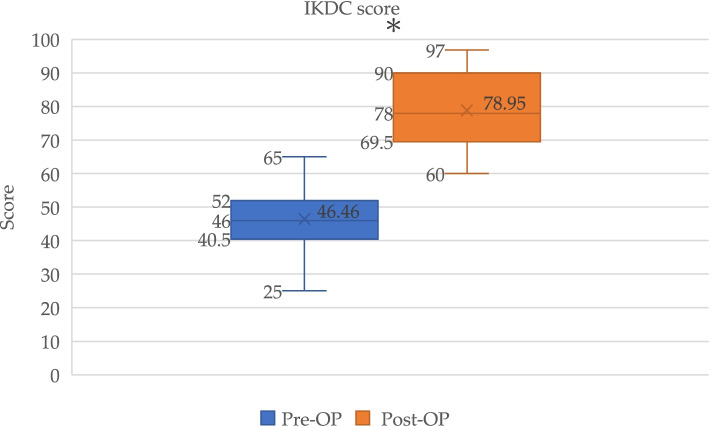
Box plot showed IKDC score for PCL reconstruction pre-operatively (Pre-Op) and post-operatively (Post-Op). *Significant (*p* < 0.0001) improvement in IKDC score

**Fig. 3:**
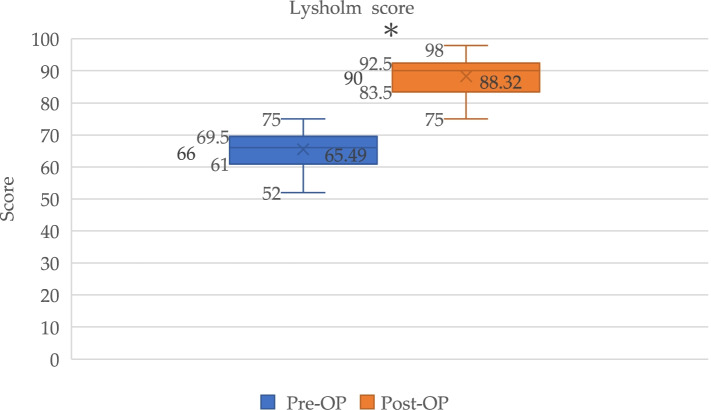
Box plot showed Lysholm scores for PCL reconstruction pre-operatively (Pre-Op) and post-operatively (Post-Op). *Significant (*p* < 0.0001) improvement in Lysholm scores

**Fig. 4:**
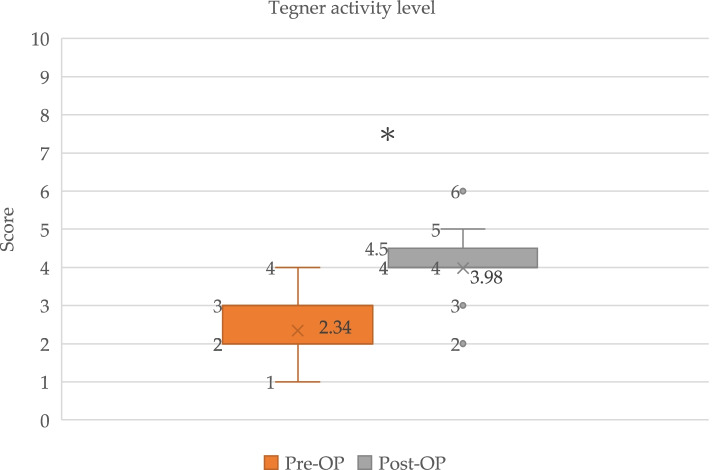
Box plot showed Tegner activity level for PCL reconstruction pre-injury, pre-operatively (Pre-Op) and post-operatively (Post-Op). *Significant (*p* < 0.0001) improvement in Tegner activity level

Functional outcomes according to the IKDC subjective score, Lysholm score, and Tegner activity level showed significant improvements between preoperative and postoperative evaluations (Figs. 2, 3, and 4).

The mean IKDC scores improved from 46.5 (95% confidence interval (CI), 43.7–49.3) preoperatively to 79.0 (95% CI, 75.7–82.2) postoperatively (*p* < 0.0001). The mean Lysholm scores also improved significantly from 65.5 (95% CI, 63.7–67.3) preoperatively to 88.3 (95% CI, 86.6–90.1) postoperatively (*p* < 0.0001). The Tegner activity level similarly improved significantly from 2.3 (95% CI, 2.1–2.5) preoperatively to 4.0 (95% CI, 3.7–4.3) postoperatively (*p* < 0.0001) (Table 4). The clinically relevant value determined using MCID showed a change in functional outcome exceeding the threshold of improvement. All patients (100%) showed a ΔLysholm score exceeding 8.9; 35 of 41 patients (85.4%) showed a ΔTegner activity score exceeding 0.5; and 34 of 41 patients (82.9%) had a ΔIKDC score exceeding 16.8. Regarding the PASS, none of the patients had an IKDC score exceeding 75.9 preoperatively, whereas 27 of 41 patients (65.9%) had a score of more than 75.9 postoperatively.

**Table 4:** Functional outcomes

Score	Pre-operative (Mean, 95% confidence interval)	Post-operative (Mean, 95% confidence interval)	*p*-value
IKDC	46.5 (43.7–49.3)	79.0 (75.7–82.2)	< 0.0001
Lysholm	65.5 (63.7–67.3)	88.3 (86.6–90.1)	< 0.0001
Tegner	2.3 (2.1–2.5)	4.0 (3.7–4.3)	< 0.0001

Of all the patients, 21 patients (51.2%) had returned to their preinjury level of activity at the last follow-up. Seventeen patients (41.5%) returned to lower preinjury activity levels by 1 level, and 3 (7.3%) patients returned to lower preinjury activity levels by 2 levels at the last follow-up. In assessment of osteoarthritis deterioration, grade I radiographic osteoarthritis was found during the follow-up period in 5 patients (12.2%), who had no evidence of osteoarthritis at the time of injury. At the last follow-up, examination showed 36 patients (87.8%) had no significant joint translation, and 5 patients (12.2%) had grade I instability according to the Petrie and Harner classification. Examination revealed negative posterior sag sign among all the patients. None of the patients had symptoms of instability during daily activity.

Before operation, 30 patients (73.2%) had a difference of 15° in flexion deficit between the healthy side and affected side. Eight patients (19.5%) had an extension deficit greater than 15°. At the last follow-up, 38 patients (92.7%) reached range of motion with smaller than 5° difference in full extension or in full flexion. Three patient (7.3%) had 10°-25° deficits in flexion.

No rerupture or other major perioperative complications were reported during the follow-up period. Minor complications occurred in 1 patient (2.4%) with superficial infection over the graft donor site, and the patient received debridement surgery.

## Discussion

To date, there is a paucity of studies reporting the outcomes of patients over the age of 40 years with PCL reconstruction. In our study, knee stability was restored in all patients aged 40 years or older with failed conservative treatment after receiving PCL reconstruction. In our series, patients receiving PCL reconstruction surgery showed significant improvements in the IKDC score, Lysholm score, and Tegner activity level, while surgery did not increase the risk of knee arthritis deterioration. No major complications were noted with a minimum follow-up time of 2 years. Approximately half of the patients returned to activities at their preinjury level.

Several studies on conservative treatment for isolated PCL injury have reported good subject and functional outcomes [1–3], but the mean age of the patients in these studies ranged from 22 to 31 years. There is a paucity of research focusing on conservative treatment for older patients with PCL injury, and age-related alterations in the ligament healing process, such as decreased healing potential, the declining function of mesenchymal stem cells, and decreased structural organization with age should considered. Stolzing et al. [21] found that the quality and quantity of human mesenchymal stem cells (hMSCs), which contribute to regeneration of various connective tissues, significantly decrease with age. However, more evidence is still needed to verify whether these age-related alterations in elderly patients result in poorer clinical and functional outcomes than observed in younger patients with PCL injuries treated nonoperatively.

With improvements in anesthesia, surgical techniques, instrumentation, and rehabilitation programs, surgeons should re-evaluate the benefits of surgical intervention in patients and adjust the indications for surgery according to recent evidence-based studies. In the present study, patient-reported outcome improvements were noted in the IKDC score (from 46.5 preoperatively to 79.0 postoperatively), Lysholm score (from 65.5 to 88.3), and Tegner activity score (from 2.3 to 4.0). Similar results were reported in previous studies that did not conduct age-subgroup analyses [7–13, 22]. A study by Belk et al. [7] that included a total of 132 patients with a mean age at the time of surgery of 31.6 years undergoing PCL reconstruction with autograft reported that patients achieved an improvement of 20 in the IKDC score, 22.7 improvement in the Lysholm score, and 3.9 improvement in the Tegner activity score. Comparing the results of these studies, it may be concluded that older patients with PCL injury can receive the same benefits from operative therapy that younger patients enjoy.

In our study, approximately half (51.2%) of the patients were able to return to activities at their preinjury level. This result is lower than those of previous studies that included patients younger in age or highly active athletes. Rauck et al. [23] reported a high rate (79%) of return to sport, overall patient satisfaction, and restoration of function, with good functional scores, after PCL reconstruction in 14 athletes with a mean age of 27.5 years (range 17–43). A study by Song et al. [12] enrolled 36 patients with a mean age of 37 years that received the transtibial technique and 30 patients with a mean age of 35 years that received tibial inlay PCL reconstruction. In their study, 21 patients (58.3%) in the transtibial group and 19 patients (63.3%) in the tibial inlay group were able to return to preinjury levels of sports activity. However, Devitt et al. [24] reviewed a combined 14 studies that reported on 523 patients with a mean age of 30.2 years and that received isolated PCL reconstruction. The results revealed a significant improvement in functional outcome scores, but a low rate (44% (95% CI, 23%-66%)) of return to preinjury level among the pooled patients. These findings provide essential information when counseling patients about realistic expectations prior to surgical intervention.

Although numerous studies have reported that nonoperative treatment of PCL injury is associated with an increased incidence of degenerative arthritis [4, 5], whether undergoing PCL reconstruction will prevent osteoarthritis progression compared with nonoperative treatment is still unknown. In this study, arthroscopic findings showed that 97.7% of patients had osteochondral lesions, and 18 (43.9%) patients showed stage I degenerative changes according to the Ahlbäck classification at the time of the preoperative radiographic study. There were no cases of osteoarthritis deterioration among these stage I osteoarthritis patients, but 5 (12.2%) patients without osteoarthritis had developed up to stage I degenerative changes at the last postoperative visit. This was also noted in most previous studies, and thus, it is difficult to distinguish whether the osteoarthritis was due to the initial trauma or to surgical intervention [22]. Hence, further high-quality randomized controlled trials focusing on comparing the incidence of osteoarthritis degeneration between patients treated with surgical reconstruction and those treated with conservative therapy are needed.

In our study, 48.8% of patients had associated meniscal lesion and half of these patients suffered from medial side tear during arthroscopic examination. This could partially explain limitation of extreme flexion (73.2% of patients) and extension (19.5% of patients) before operation, and range of motion improved greatly after meniscal tear being treated with either partial meniscectomy or meniscal repair. Zhang et al. [25] reported PCL injury resulted in radial displacement of the medial meniscus which may lead to degenerative changes of meniscus. A study by Gao et al. [26] revealed human cadaveric knee with PCL transection had a higher strain on whole medial meniscus. Pearsall et al. [27] reported meniscal strain increased in PCL injured knee and decreased after PCL reconstruction. According to these studies, early intervention with PCL reconstruction plays an important role in reducing meniscal strain and subsequent degeneration.

There has been increasing recognition that the health status, type and level of activity of the older population have changed significantly in many parts of the world over the past few decades. This has led to the development of alternative concepts to offer more comprehensive management when surgeons perform preoperative assessments. Physiological age is more important than chronological age in PCL-deficient patients. To provide appropriate treatment, surgeons should take patients’ expectations, lifestyle, and activity level before injury into consideration.

The limitations of this study include the absence of a control group, the small number of patients, the short observation period, and the lack of objective information on KT-1000 arthrometer and stress radiographs compared with the healthy side. Further studies with a comparison between young and older patients are needed to clarify the clinical and functional outcomes of PCL reconstruction in different age groups. Additionally, this is a retrospective study with heterogeneous surgical techniques. A longer follow-up period is required to observe the incidence of later complications or further arthritis deterioration. Lastly, the patients underwent conservative treatment before surgery. Although it failed, there is a possibility that conservative treatment contributed part of the results. Nonetheless, subsequent operative intervention after failed non-operative management is in line with current treatment strategy for PCL injury.

## Conclusions

PCL reconstruction is a reliable surgery for middle-aged patients suffering from persistent instability even after failed conservative treatment with significant improvement in patient-reported outcomes that exceeded MCID in the majority of patients, restoration of subjective instability, and approximately half of the patients returned to preinjury activity levels.

## Data Availability

The data presented in this study are available on request from the corresponding author. The data are not publicly available due to privacy.

## Abbreviations

PCL: Posterior cruciate ligament
ACL: Anterior cruciate ligament
MRI: Magnetic resonance imaging
IKDC: International knee documentation committee
MCID: Minimal clinically important difference
PASS: Patient acceptable symptom state
CI: Confidence interval

## References

[1] Shelbourne KD, Clark M, Gray T (2013). Minimum 10-year follow-up of patients after an acute, isolated posterior cruciate ligament injury treated nonoperatively. Am J Sports Med.

[2] Shelbourne KD, Muthukaruppan Y (2005). Subjective results of nonoperatively treated, acute, isolated posterior cruciate ligament injuries. Arthroscopy.

[3] Shelbourne KD, Davis TJ, Patel DV (1999). The natural history of acute, isolated, nonoperatively treated posterior cruciate ligament injuries. A prospective study. Am J Sports Med.

[4] Van de Velde SK, Bingham JT, Gill TJ, Li G (2009). Analysis of tibiofemoral cartilage deformation in the posterior cruciate ligament-deficient knee. J Bone Joint Surg Am.

[5] Strobel MJ, Weiler A, Schulz MS, Russe K, Eichhorn HJ (2003). Arthroscopic evaluation of articular cartilage lesions in posterior-cruciate-ligament-deficient knees. Arthroscopy.

[6] Amis AA, Bull AM, Gupte CM, Hijazi I, Race A, Robinson JR (2003). Biomechanics of the PCL and related structures: posterolateral, posteromedial and meniscofemoral ligaments. Knee Surg Sports Traumatol Arthrosc.

[7] Belk JW, Kraeutler MJ, Purcell JM, McCarty EC (2018). Autograft Versus Allograft for Posterior Cruciate Ligament Reconstruction: An Updated Systematic Review and Meta-analysis. Am J Sports Med.

[8] Lind M, Nielsen TG, Behrndtz K (2018). Both isolated and multi-ligament posterior cruciate ligament reconstruction results in improved subjective outcome: results from the Danish Knee Ligament Reconstruction Registry. Knee Surg Sports Traumatol Arthrosc.

[9] Song JG, Kim HJ, Han JH, Bhandare NN, Shetty GM, Kang SB, Song YW, Nha KW (2015). Clinical Outcome of Posterior Cruciate Ligament Reconstruction With and Without Remnant Preservation. Arthroscopy.

[10] Ansari AS, Dennis BB, Horner NS, Zhu M, Brookes C, Khan M, Grant JA (2019). Influence of Graft Source on Postoperative Activity and Joint Laxity in Posterior Cruciate Ligament Reconstruction: A Systematic Review. Arthroscopy.

[11] Yoon KH, Kim EJ, Kwon YB, Kim SG (2019). Minimum 10-Year Results of Single- Versus Double-Bundle Posterior Cruciate Ligament Reconstruction: Clinical, Radiologic, and Survivorship Outcomes. Am J Sports Med.

[12] Song EK, Park HW, Ahn YS, Seon JK (2014). Transtibial versus tibial inlay techniques for posterior cruciate ligament reconstruction: long-term follow-up study. Am J Sports Med.

[13] LaPrade RF, Cinque ME, Dornan GJ, DePhillipo NN, Geeslin AG, Moatshe G, Chahla J (2018). Double-Bundle Posterior Cruciate Ligament Reconstruction in 100 Patients at a Mean 3 Years' Follow-up: Outcomes Were Comparable to Anterior Cruciate Ligament Reconstructions. Am J Sports Med.

[14] Brown CA, McAdams TR, Harris AH, Maffulli N, Safran MR (2013). ACL reconstruction in patients aged 40 years and older: a systematic review and introduction of a new methodology score for ACL studies. Am J Sports Med.

[15] Desai N, Bjornsson H, Samuelsson K, Karlsson J, Forssblad M (2014). Outcomes after ACL reconstruction with focus on older patients: results from The Swedish National Anterior Cruciate Ligament Register. Knee Surg Sports Traumatol Arthrosc.

[16] Toanen C, Demey G, Ntagiopoulos PG, Ferrua P, Dejour D (2017). Is There Any Benefit in Anterior Cruciate Ligament Reconstruction in Patients Older Than 60 Years?. Am J Sports Med.

[17] Weng CJ, Yeh WL, Hsu KY, Chiu CH, Chang SS, Chen AC, Chan YS (2020). Clinical and Functional Outcomes of Anterior Cruciate Ligament Reconstruction With Autologous Hamstring Tendon in Patients Aged 50 Years or Older. Arthroscopy.

[18] Harner CD, Höher J (1998). Evaluation and treatment of posterior cruciate ligament injuries. Am J Sports Med.

[19] Harris JD, Brand JC, Cote MP, Faucett SC, Dhawan A (2017). Research Pearls: The Significance of Statistics and Perils of Pooling. Part 1: Clinical Versus Statistical Significance. Arthroscopy.

[20] Muller B, Yabroudi MA, Lynch A, Lai CL, van Dijk CN, Fu FH, Irrgang JJ (2016). Defining Thresholds for the Patient Acceptable Symptom State for the IKDC Subjective Knee Form and KOOS for Patients Who Underwent ACL Reconstruction. Am J Sports Med.

[21] Stolzing A, Jones E, McGonagle D, Scutt A (2008). Age-related changes in human bone marrow-derived mesenchymal stem cells: consequences for cell therapies. Mech Ageing Dev.

[22] Kim YM, Lee CA, Matava MJ (2011). Clinical results of arthroscopic single-bundle transtibial posterior cruciate ligament reconstruction: a systematic review. Am J Sports Med.

[23] Rauck RC, Nwachukwu BU, Allen AA, Warren RF, Altchek DW, Williams RJ (2019). Outcome of isolated posterior cruciate ligament reconstruction at mean 6.3-year follow up: a consecutive case series. Phys Sportsmed.

[24] Devitt BM, Dissanayake R, Clair J, Napier RJ, Porter TJ, Feller JA, Webster KE (2018). Isolated Posterior Cruciate Reconstruction Results in Improved Functional Outcome but Low Rates of Return to Preinjury Level of Sport: A Systematic Review and Meta-analysis. Orthop J Sports Med.

[25] Zhang C, Deng Z, Luo W, Xiao W, Hu Y, Liao Z, Li K, He H (2017). Rupture of posterior cruciate ligament leads to radial displacement of the medial meniscus. BMC Musculoskelet Disord.

[26] Gao SG, Zhang C, Zhao RB, Liao Z, Li YS, Yu F, Zeng C, Luo W, Li KH, Lei GH (2013). Effect of partial and complete posterior cruciate ligament transection on medial meniscus: A biomechanical evaluation in a cadaveric model. Indian J Orthop.

[27] Pearsall AWt, Hollis JM (2004). The effect of posterior cruciate ligament injury and reconstruction on meniscal strain. Am J Sports Med.

